# Biventricular Failure due to Stress Cardiomyopathy after Pericardiectomy for Constrictive Pericarditis

**DOI:** 10.1155/2013/106757

**Published:** 2013-11-28

**Authors:** Elliott M. Groves, Jin Kyung Kim

**Affiliations:** ^1^Division of Cardiology, Department of Medicine, University of California at Irvine, 333 City Blvd West, Suite 400, Orange, CA 92868, USA; ^2^Edwards Lifesciences Center for Advanced Cardiovascular Technology, Department of Bioengineering, University of California at Irvine, 5200 Engineering Hall, Irvine, CA 92697, USA; ^3^UC Irvine Medical Center, 101 The City Drive, Orange, CA 92868, USA

## Abstract

*Importance*. Constrictive pericarditis is a rare clinical entity that frequently necessitates surgical intervention. Here we present a case of biventricular failure due to stress cardiomyopathy after pericardiectomy. This is an extremely rare complication that is not well described and does not have a definitive mechanism. *Observations*. A 40-year-old Ecuadorian woman who was found to have constrictive pericarditis due to *Mycobacterium tuberculosis *infection was referred to our institution. The presence of constrictive pericarditis was confirmed by echocardiography, computed tomography, magnetic resonance imaging, and cardiac catheterization. Following pericardiectomy, the patient developed biventricular failure consistent with stress cardiomyopathy (Takotsubo cardiomyopathy), based on the echocardiographic assessment of the ventricles, which demonstrated an akinetic apex and hyperactive base in both ventricles, the absence of significant epicardial coronary atherosclerosis, and prompt normalization of the cardiac function after intensive medical therapy. *Conclusions and Relevance*. Biventricular failure in the form of stress cardiomyopathy after pericardiectomy in the manner presented here has not been previously described in the literature. While postulations as to the cause of single ventricle dysfunction have been described, the exact mechanism is unclear and current theories do not explain the clinical features in this case of stress cardiomyopathy after pericardiectomy.

## 1. Introduction

Constrictive pericarditis is a relatively rare but well-described clinical entity [1]. It is a postinflammatory disorder in which the loss of pericardial elasticity results in a restriction of diastolic filling [2]. Etiologies vary and include infectious causes, uremia, chest radiation, collagen vascular diseases, idiopathic and postoperative processes [2, 3]. Definitive treatment is surgical and involves removal of the pericardium [4]. Right ventricular dysfunction is a rare but known complication in the postoperative period [4, 5]. However, biventricular dysfunction, to our knowledge, has only been reported once in the literature and without the presentation of the echocardiographic images; thus the nature of the dysfunction could not be assessed [2]. Here we present a unique case of a woman with a history of tuberculosis, who suffered severe biventricular dysfunction in the form of stress cardiomyopathy (also called Takotsubo cardiomyopathy) after pericardiectomy was performed as a treatment for her constrictive pericarditis.

## 2. Case

A 40-year-old woman originally from Ecuador was referred to our institution for evaluation of progressive dyspnea on exertion. Two months prior to her presentation, she was treated at an outside hospital for presumed community acquired pneumonia with symptoms of dyspnea on exertion, fevers, chills, and productive cough. After not seeing measurable improvement, the patient then presented to the emergency department at another institution where a transthoracic echocardiogram (TTE) revealed that the patient had a large pericardial effusion with tamponade physiology and a normal left ventricular ejection fraction (LVEF) of 60%. Pericardiocentesis was performed with 700 cc of fluid removed, which grew *Mycobacterium tuberculosis* (TB). The patient's purified protein derivative (PPD) skin test was positive. The patient was started on anti-TB therapy and discharged.

**Figure 1:**
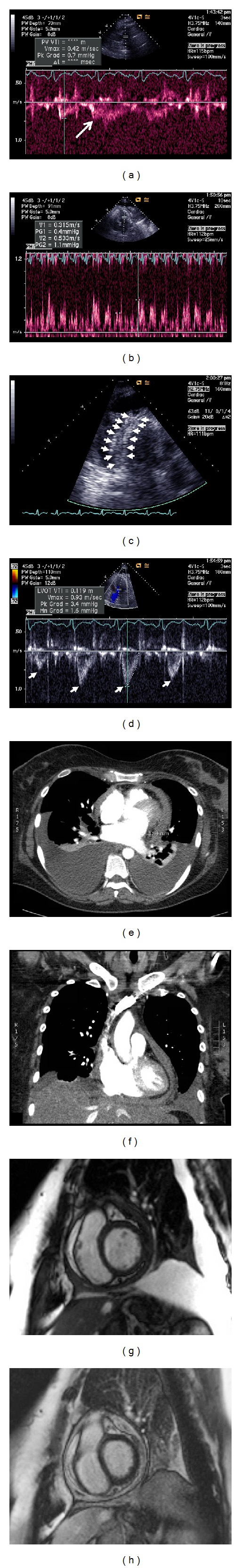
Imaging findings consistent with constriction. Initial transthoracic echocardiography shows Doppler spectral revealing a presystolic flow (white arrow) through the pulmonic valve due to premature opening of the valve (a), exaggerated respiratory variation of the mitral valve inflow (b), an apical 3-chamber view of the thickened pericardium (outlined by arrows) around the inferolateral/posterior wall of the left ventricle (c), and exaggerated left ventricular outflow tract flow variation (arrows) (d). Cardiac CT with contrast demonstrates axial (e) and coronal (f) images of the markedly thickened pericardium measuring 13.4 mm and pleural effusion. Cardiac MR also demonstrates the thickened pericardium in the T2-weighted bright blood imaging (g) and the intense late gadolinium enhancement of the pericardium without myocardial involvement (h).

She continued to complain of persistent dyspnea on exertion and thus was referred to our institution for a repeat of TTE at the outpatient cardiac diagnostic center. TTE revealed the rare presystolic flow through pulmonic valve, excessive variation in the mitral valve and aortic valve spectral Doppler velocities, and markedly thickened pericardium (Figures 1(a)–1(d)), consistent with constriction [6]. The study also demonstrated septal bounce, small pericardial effusion, and pleural effusion. The patient was admitted for further evaluation. On admission, the patient's vital signs showed a temperature of 37 degrees Celsius, a blood pressure of 108/70, a heart rate of 117, and respiratory rate of 20 with an oxygen saturation of 99% in room air. Her exam revealed a young woman in mild distress with 11 cm internal jugular vein distension, decreased breath sounds at lung bases bilaterally and scant bibasilar crackles, regular tachycardia, and bilateral lower extremity edema. Her BNP was 124, CRP was 5.1, and WBC count was 5.9 with a normal differential. Her complete metabolic panel was unremarkable. The patient underwent a left and right heart catheterization due to the suspicion of constrictive pericarditis. A coronary angiogram demonstrated no obstructive epicardial coronary artery disease. The right heart catheterization revealed elevated right atrial pressure, right ventricular end diastolic pressure, and an elevated pulmonary capillary wedge pressure. Simultaneous right and left ventricular pressure tracings demonstrated discordant respiratory variation consistent with constrictive pericarditis. A computed tomography (CT) scan of her chest with contrast confirmed marked thickening of both the parietal and pleural pericardial surfaces, measuring up to 13.4 mm in thickness, and a large right-sided pleural effusion (Figures 1(e) and 1(f)). Cardiac magnetic resonance imaging (MRI) with and without contrast showed diffuse pericardial thickening (Figure 1(g)), which showed intense delayed gadolinium contrast enhancement (Figure 1(h)) that did not involve the myocardium. Additionally, there was a greater interventricular septal bounce during diastole and inspiration, consistent with chronic inflammatory constrictive pericarditis.

Due to her worsening symptoms and diagnosis of constrictive pericarditis, she was referred to cardiothoracic surgery at our institution for pericardiectomy. The surgery was without incident and the surgical samples were sent for pathology. While being negative for AFB by concentrated smear and culture, the stripped pericardium showed noncaseating granulomas, extensive fibrosis, and severe lymphohistiocytic infiltrate.

**Figure 2:**
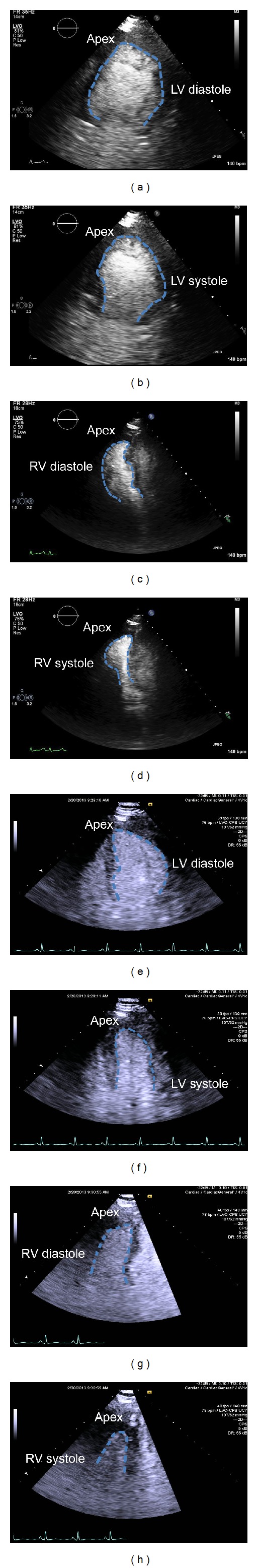
Postpericardiectomy transthoracic echocardiograms illustrating stress cardiomyopathy and recovery. The end diastolic and end systolic ((a) and (b), resp.) left ventricle in apical 2-chamber views and the end diastolic and end systolic ((c) and (d), resp.) right ventricle in apical 4-chamber views illustrating the akinetic apical and midregional wall motion abnormalities and hyperdynamic contractile base of both ventricles, consistent with stress cardiomyopathy, on postoperative day 1. Postdischarge echocardiography 7 weeks later shows normalized wall motion and function of the left ((e) and (f)) and right ventricles ((g) and (h)) in end diastole and end systole, respectively. The endocardial border is outlined in blue dashed line. The apex of the left and right ventricles is oriented to the top of each panel as marked.

In the immediate postoperative period, the patient began to develop signs and symptoms of central volume overload, heart failure, and hypotension. An electrocardiogram (ECG) at this time showed sinus tachycardia with nonspecific ST-T abnormalities with poor R wave progression, new from prior ECGs. A subsequent TTE on postoperative day one showed significant changes from the prior study, with severely decreased biventricular dysfunction. The LVEF was severely depressed at 26%, with early evidence of elevated filling pressure within the left ventricle, indicated by the time difference between pulmonary vein (Ar) duration and mitral A-wave duration (Ar-A) of positive 46 ms. For the right ventricle, fractional area change (FAC) was abnormally low at 29%, with reduced tricuspid annular plane systolic excursion (TAPSE) distance of 0.96 cm. There was also moderate tricuspid regurgitation, enlarged right atrium (area 27 cm^2^), and moderately elevated pulmonary artery systolic pressure of 41 mmHg. There was akinesis of the entire apical and midventricular segments of both the right and left ventricles (Figures 2(a)–2(d)) with hypercontractile base. Findings were consistent with stress cardiomyopathy.

While in critical condition postoperatively, the patient had episodes of atrial fibrillation and atrial tachycardia, for which she was chemically cardioverted with intravenous amiodarone, with subsequent conversion to sinus rhythm. Initially, the patient required multiple vasopressors and inotropes (epinephrine, norepinephrine, milrinone, and dopamine drip) to maintain her blood pressure and cardiac output, while gentle diuresis was achieved carefully for the symptoms of heart failure. Once the patient was hemodynamically stable, she was placed on appropriate oral medical therapy, including carvedilol and furosemide, followed by gradual clinical improvement and was able to be discharged on postoperative day 10. Due to decreased urine output and hypotension, an angiotensin-converting-enzyme inhibitor or angiotensin receptor blocker was not initiated at the time. A posthospital TTE 7 weeks after discharge showed normalized function of both the right and left ventricles (Figures 2(e)–2(h)).

## 3. Discussion

Here we describe a rare case of severe biventricular dysfunction in the form of stress cardiomyopathy following a pericardiectomy. The patient had an abrupt decrease in her left and right ventricular function following her surgery, manifested by symptoms of heart failure and biventricular failure on TTE with regional wall motion abnormalities consistent with stress cardiomyopathy.

To our knowledge, there are no definitive studies to illustrate the mechanism of biventricular dysfunction after pericardiectomy. However, several hypotheses as to the etiology of ventricular dysfunction have been considered. The first involves myocardial atrophy and the effect of the increase in venous return. It is felt that patients with constrictive pericarditis over time develop a reduction in myocardial mass due to the reduced diastolic volumes [4]. Therefore, when the pericardium is removed and the ventricle is exposed to a greater volume, as happens postoperatively when the right atrial pressure drops significantly, dilation occurs and cardiac output is decreased. However, due to the fact that this is not a disease of the myocardium, recovery of the myocardium is possible and quite often this occurs, as illustrated by a limited series of cases of postoperative right ventricular dysfunction [5]. A second theory involves a change in coronary perfusion as a result of a rapid increase in ventricular wall tension [4]. As the right and left ventricles expand after pericardiectomy, wall tension increases and perfusion is affected. Additional explanations include intraoperative trauma to the myocardium and involvement of the myocardium in the inflammatory disease [7].

Each of these explanations cannot fully explain the regional wall motion abnormalities manifested in our patient, consistent more with stress or Takotsubo cardiomyopathy. An increase in venous return to the right ventricle would certainly explain the overall right ventricular dysfunction but would not fully explain the pattern of regional biventricular dysfunction, particularly if the volume delivered to the left ventricle was not dramatically increased. Changes in coronary perfusion could explain the findings; however, the regional wall motion abnormalities in multiple vascular territories would be unlikely. Finally, the surgeon did not report intraoperative trauma, and the MRI excluded myocardial involvement.

In conclusion, presented here is an interesting and extremely rare case of postpericardiectomy biventricular failure due to stress cardiomyopathy.
